# Rare intraparotid gland localization of Kimura's disease: A case report

**DOI:** 10.1016/j.ijscr.2025.111280

**Published:** 2025-04-07

**Authors:** Trigui Majdi, Werda Majd, Sellami Moncef, Kharrat Rania, Charfeddine Ilheme, Majd Werda

**Affiliations:** a ENT – Head and Neck Department, Habib Bourguiba University Hospital, Sfax, Tunisia; b ENT Department, Habib Bourguiba University Hospital, Sfax, Tunisia

**Keywords:** Kimura's disease, Parotid, Surgery, Head and neck

## Abstract

**Introduction and importance:**

Kimura's disease, a rare chronic inflammatory disorder of uncertain etiology, predominantly affects Asian males. Its occurrence in non-Asian individuals is uncommon, often leading to diagnostic delays. We present a North African case to underscore its global relevance.

**Case presentation:**

A 31-year-old male presented with a 6-year history of a firm, mobile parotid mass. MRI identified a nodular lesion in the superficial parotid lobe. Histopathology confirmed Kimura's disease. The patient underwent exofacial parotidectomy with no recurrence.

**Clinical discussion:**

Kimura's disease must be distinguished from malignant or infectious parotid masses. Surgical excision is curative for localized disease; corticosteroids address systemic involvement. Renal complications (seen in 50 % of cases) require monitoring. Radiotherapy/immunosuppressants remain adjunctive.

**Conclusion:**

This case emphasizes including Kimura's disease in differential diagnoses of parotid masses, regardless of ethnicity. Its diagnosis is based on histopathologic findings, and treatment should be individualized.

## Highlights

• First reported North African case of Kimura's disease with isolated parotid gland involvement, expanding the known demographic spectrum.
• Multimodal diagnostic approach: Characteristic MRI findings (e.g., ADC ratio 0.87) and histopathology (eosinophilic infiltration, IgE deposits) confirmed diagnosis.
• Definitive surgical management: Exofacial parotidectomy with nerve preservation resulted in recurrence-free survival at 3-year follow-up.
• Critical diagnostic insights: Highlights the role of histopathology in differentiating Kimura's disease from malignancies (e.g., lymphoma) in non-endemic populations.
• Therapeutic implications: Supports surgery as first-line for localized disease, with corticosteroids reserved for systemic involvement.

## 1 Introduction

Kimura's disease, or eosinophilic lymphogranuloma, is a rare inflammatory condition with an unclear etiopathogenesis [1]. Initially associated with a pseudo-tumoral process affecting the vascular endothelium, recent pathophysiological understanding of this disorder defines it as a reactive allergic or autoimmune process involving blood vessels, lymphocytes, and eosinophils.

This condition predominantly affects individuals of East Asian origin [2]. We report a case of parotid gland localization of Kimura's disease in a patient from North Africa.

Informed consent was obtained from the patient for the publication of this case report, including all accompanying images and clinical details. This research received no specific grant from any funding agency in the public, commercial, or not-for-profit sectors. The authors declare that they have no conflicts of interest regarding the publication of this case report. This case report has been reported in accordance with the SCARE criteria [3].

## 2 Case presentation

The patient was a 31-year-old male, non-smoker and non-drinker, with no relevant medical history. He had a parotid swelling that had been present for six years, without associated facial asymmetry.

There were no signs of salivary colic or salivary hernia.

Clinical examination revealed a superficial right parotid mass, measuring 5 cm in diameter, located anterior to the tragus. It was firm to palpation, with regular contours and mobile in relation to both superficial and deep planes. The overlying skin appeared healthy. Notably, there was no facial nerve dysfunction or impairment of saliva production. No pyosialia or salivary duct hemorrhage was noted, and the Stensen duct was intact.

There were no palpable lymphadenopathies, and no swelling of the contralateral parotid gland.

**Fig. 1:**
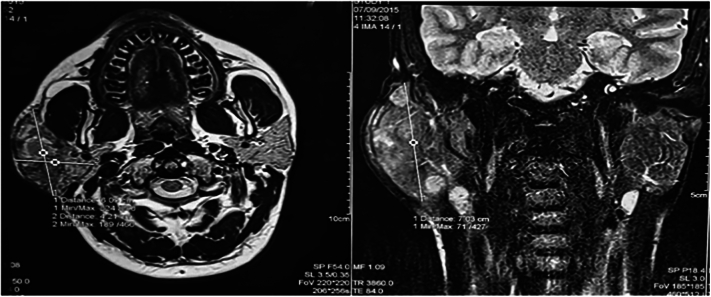
MRI showing axial and coronal reconstructions of a parotid mass exhibiting hypointensity on T1 and hyperintensity on T2.

An MRI of the parotid **(**Fig. 1**)** showed a nodular mass in the superficial lobe of the right parotid with irregular contours, measuring 60 × 42 mm axially and 70 mm in height. The mass demonstrated hypointensity on T1-weighted images and hyperintensity on T2-weighted images, with heterogeneous signal and diffuse heterogeneous contrast enhancement. There were also multiple non-suspicious bilateral jugulocarotid lymphadenopathies. The ADC ratio was 0,87. The biological workup revealed mild leukocytosis (6800/μL) with marked hypereosinophilia (740/μL), elevated IgE levels (680 IU/mL), and normal IgG levels (0.42 g/L). Renal function was preserved (urea: 4.2 mmol/L, creatinine: 78 μmol/L), and the ionogram showed no abnormalities.

A fine needle aspiration (FNA) was performed and showed signs of non-specific inflammation, without evidence of malignancy.

An exofacial parotidectomy was performed, with a 5 cm specimen resected. It appeared to be an inflammatory tumor with a firm consistency.

The intraoperative frozen section revealed non-specific inflammatory tissue with eosinophilic hyperplasia.

**Fig. 2:**
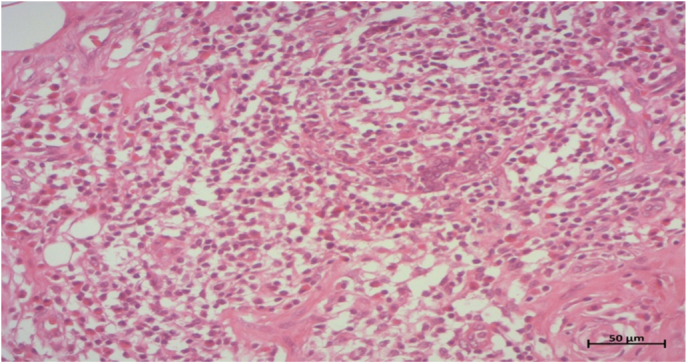
Histopathological examination of the parotid mass demonstrating lymphocytic infiltrate rich in eosinophils, consistent with Kimura's disease.

The definitive histopathological examination confirmed the diagnosis of Kimura's disease localized to the parotid, showing a lymphocytic infiltrate rich in eosinophils **(**Fig. 2**)**.

The postoperative course was uneventful. The bilateral centimetric jugulocarotid lymphadenopathies resolved spontaneously within the first postoperative month under surveillance, with normalization of eosinophil count (180/μL) and IgE (220 IU/mL). Surveillance was quarterly for the first year, then biannually. The patient had no recurrence or secondary lesions during 3 years of follow-up.

## 3 Discussion

Kimura's disease was first described in Japan in 1948 as an “unusual granuloma associated with hyperplastic changes in lymphoid tissue” [4]. This condition predominantly affects males, with a male-to-female ratio of 3/7, and typically occurs in individuals aged 20 to 40 years.

Clinically, it is characterized by a triad of features: cutaneous involvement, glandular involvement, and renal involvement [5,6].

Notably, the diagnosis hinges on excluding mimics such as angiolymphoid hyperplasia with eosinophilia (ALHE) (superficial lesions without renal disease) and lymphoproliferative disorders (e.g., lymphoma), which lack the eosinophilic infiltration and IgE elevation typical of Kimura's disease [7,8].

Cutaneous involvement consists of ill-defined subcutaneous nodules, which may vary in consistency from firm to soft. These lesions are often adherent to the overlying skin, which may appear normal or slightly brownish in color [1].

In approximately half of the cases, these lesions are multiple, and they predominantly affect the cervicofacial region [9], including the peri-auricular area and lateral neck. However, other locations have also been described.

Glandular involvement primarily affects the salivary glands, particularly the parotid gland, as seen in our case. This involvement is almost always associated with inflammatory lymphadenopathy [1].

While the lesions associated with Kimura's disease do not carry a risk of malignant degeneration, the prognosis is primarily influenced by renal involvement, which occurs in 50 % of cases and manifests as proteinuria or nephrotic syndrome due to membranous glomerulonephritis [10].

The treatment of glandular localization is primarily surgical, with complete and wide resection being crucial to avoid recurrence, which occurs in 15 to 40 % of cases [1,6,11].

High-dose corticosteroids (e.g., prednisone 0.5–1 mg/kg/day for 2–4 weeks, followed by a gradual taper over 2–3 months) are the most commonly used medical treatment. They are indicated in cases of multifocal disease, renal involvement, or surgically inaccessible lesions, where they reduce inflammation and eosinophilic infiltration [1].

Other treatments, such as chemotherapy with 5-fluorouracil or radiotherapy (25 to 30 Gray), have been described, although their benefits have not been definitively proven [6]. Emerging therapies show promise for refractory cases; Cyclosporine A (3–5 mg/kg/day) targets T-cell-driven eosinophilia, with reported success in steroid-resistant Kimura's disease [12]. Anti-IL-5 biologics (e.g., mepolizumab 300 mg/month) have induced remission in cases with persistent hypereosinophilia [13,14].

## 4 Conclusion

Kimura's disease is a rare condition that primarily affects adult males of Asian descent. Its diagnosis is histological. Treatment typically involves surgery for glandular tumor involvement and corticosteroid therapy. The prognosis of the disease remains favorable, mainly determined by the extent of renal involvement.

## Consent

Written informed consent was obtained from the patient for the publication of this case report and accompanying images.

## Ethical approval

Ethical approval was not required for this case report, as per institutional guidelines. However, all ethical principles were adhered to in the preparation of this manuscript.

## Funding

This research did not receive any specific grant from funding agencies in the public, commercial, or not-for-profit sectors.

## Author contribution

1. **Trigui Majdi**
  o Data analysis and interpretation
  o Critical revision of the paper for intellectual content
  o Contribution to study design
2. **Werda Majd** (Principal Author)
  o Study concept and design
  o Data collection and analysis
  o Writing the paper (primary responsibility)
  o Overall coordination and supervision of the research
3. **Sellami Moncef**
  o Data collection
  o Technical or methodological support
  o Reviewing and editing the paper
4. **Kharrat Rania**
  o Data interpretation
  o Literature review and background research
  o Contribution to writing specific sections of the paper
5. **Charfeddine Ilheme**
  o Technical or logistical support
  o Reviewing and editing the paper
  o Contribution to data visualization or presentation

## Guarantor

Trigui Majdi

## Conflict of interest statement

The authors declare that they have no conflicts of interest related to this work.
